# Advances in PD-1 and CTLA-4 dual-target immunotherapy for ovarian cancer

**DOI:** 10.3389/fimmu.2025.1686532

**Published:** 2025-11-03

**Authors:** Dong-Mei Li, Kai-Ge Pei, Xiu-Zhang Yu, Ming-Rong Qie, Lan Zhong, Liang Song

**Affiliations:** ^1^ Department of Obstetrics and Gynecology, West China Second University Hospital, Sichuan University, Chengdu, China; ^2^ Key Laboratory of Birth Defects and Related Diseases of Women and Children (Sichuan University), Ministry of Education, Chengdu, China

**Keywords:** ovarian cancer, dual immune checkpoint inhibitors, PD-1, CTLA-4, neoadjuvant therapy

## Abstract

Ovarian cancer(OC) remains a major threat to women’s health, ranking among the top gynecologic malignancies in both incidence and mortality. Current clinical management continues to center on cytoreductive surgery combined with a multidisciplinary approach incorporating chemotherapy, targeted therapy, and immunotherapy. Notably, while single-agent immunotherapy has demonstrated limited efficacy in recurrent OC, recent breakthrough advances in dual-target immunotherapy have brought new hope for advanced-stage and recurrent patients. Clinical evidence indicates that programmed death-1/cytotoxic T-lymphocyte-associated protein 4 (PD-1/CTLA-4) dual immune checkpoint blockade strategies (e.g., durvalumab plus tremelimumab, nivolumab plus ipilimumab) exhibit differential therapeutic effects: durable treatment responses have been observed in recurrent/platinum-resistant advanced OC, while neoadjuvant applications have significantly improved complete resection rates. However, these therapeutic benefits demonstrate marked heterogeneity across different histological subtypes. The review of current research reveals several critical issues: first, the safety profile of dual immunotherapy requires further characterization; second, data on first-line treatment for advanced OC remain scarce; and third, optimal treatment strategies have yet to be established. Nevertheless, multiple ongoing clinical trials are paving the way for future research directions, including optimization of combination regimens and exploration of predictive biomarkers. In conclusion, despite existing challenges, dual-target immunotherapy has demonstrated clinically meaningful benefits, offering new therapeutic options for advanced and recurrent OC patients and heralding a new era of combination immunotherapy in OC treatment. Future large-scale clinical studies are warranted to further validate efficacy and establish individualized precision treatment strategies.

## 1 Introduction

Ovarian cancer (OC) ranks among the most common and lethal gynecologic malignancies worldwide (1). Owing to the absence of reliable early screening methods and its insidious clinical presentation, approximately 70% of patients are diagnosed at an advanced stage (2). Epidemiological studies report an overall 5-year survival rate of around 40%, with the 10-year survival plummeting to a mere 13% in advanced cases (3). The current therapeutic paradigm centers on cytoreductive surgery, supplemented by a multimodal approach integrating chemotherapy, targeted therapy, and immunotherapy (4). Immune checkpoint inhibitors (ICIs), now established as a cornerstone in treating various solid tumors (including cervical and endometrial cancers), have shown promise in OC. Preclinical evidence demonstrates that T-cell infiltration within ovarian tumor tissues correlates with anti-tumor immune activation at the molecular level, and this immune signature is positively associated with improved survival—lending a strong rationale for ICI-based interventions (5, 6). Contemporary immunotherapeutic strategies focus on enhancing cytotoxic immune responses to mitigate tumor burden. Nevertheless, response heterogeneity to ICIs persists across histological subtypes and individual patients, driven by factors such as tumor microenvironment composition, tumor mutational burden (TMB), immune checkpoint expression patterns, and genetic/epigenetic regulatory mechanisms. The intricate interplay of these elements ultimately dictates therapeutic outcomes (7–10). Amidst the rapid development of novel agents and combination regimens, the management of advanced and metastatic OC is witnessing a transformative shift, offering renewed hope for this historically recalcitrant disease.

In gynecologic oncology, ICIs targeting programmed death-1 (PD-1)/programmed death-ligand 1 (PD-L1) and cytotoxic T-lymphocyte-associated protein 4 (CTLA-4) have advanced from second-line to first-line treatment for advanced/recurrent cervical and endometrial carcinomas. However, OC remains refractory to ICI therapy due to its immunologically “cold” tumor phenotype (11). This immunotherapeutic resistance is characterized by TMB, inadequate immune cell infiltration, and a profoundly immunosuppressive tumor microenvironment (TME) dominated by regulatory T cells (Tregs) and myeloid-derived suppressor cells (MDSCs), coupled with limited tumor antigen expression that impedes immune recognition (7, 8).

**Figure 1:**
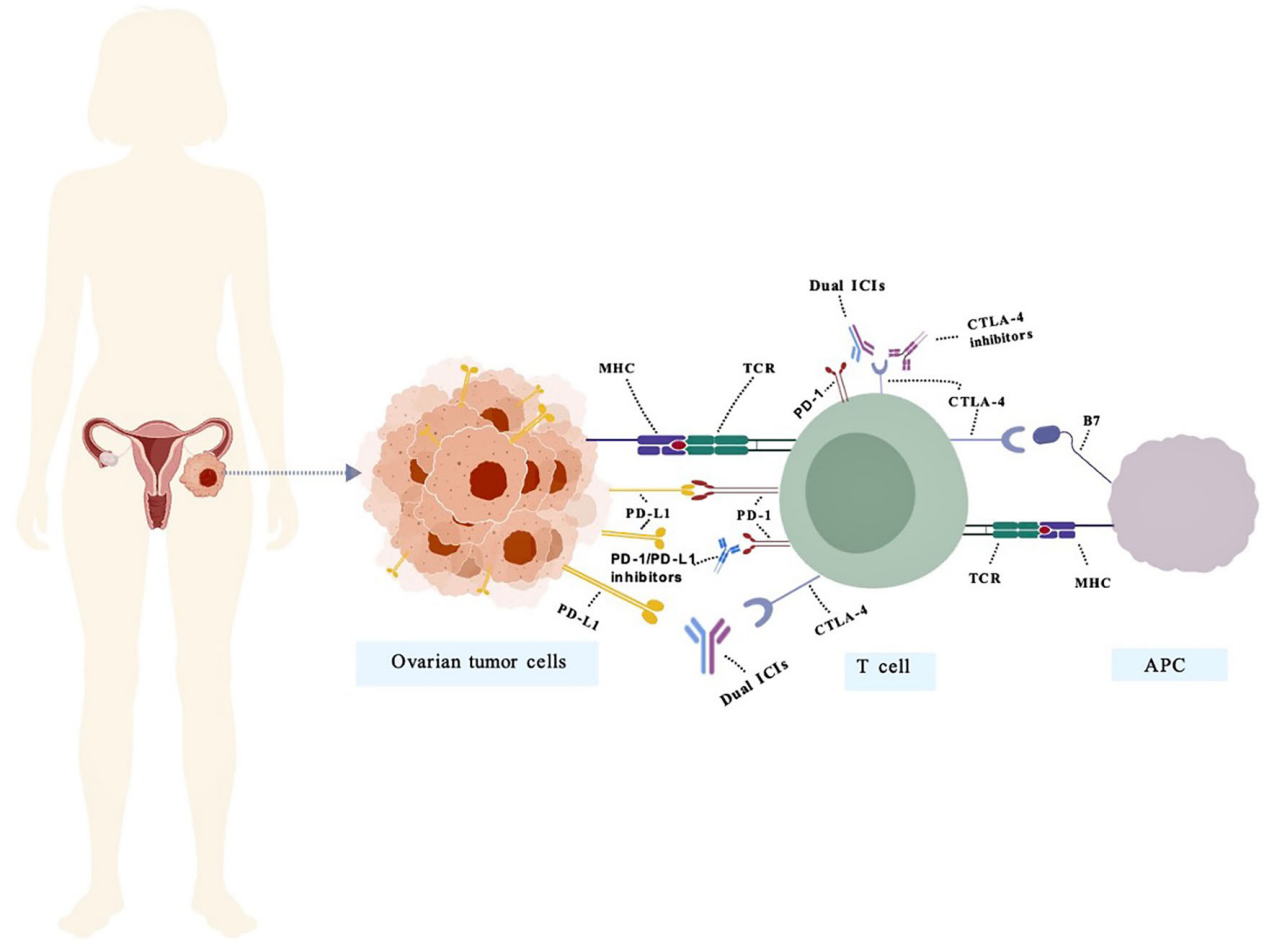
The mechanism of action of bispecific antibodies targeting PD-1/PD-L1 and CTLA-4 in OC. PD-1 negatively regulates effector T-cell activity by binding to its ligand PD-L1. Inhibitors targeting PD-1 or PD-L1 disrupt this interaction, thereby restoring T-cell function and promoting anti-tumor immunity. Representative PD-1/PD-L1 inhibitors include pembrolizumab, nivolumab, atezolizumab, and durvalumab. CTLA-4 binds to B7 molecules on antigen-presenting cells (APCs), transmitting inhibitory signals to T cells. CTLA-4 inhibitors restore immune activity by blocking this interaction. Representative CTLA-4 inhibitors include Ipilimumab and Tremelimumab. Dual ICIs mitigate immunosuppression by concurrently targeting multiple inhibitory pathways, thereby overcoming immune tolerance within the tumor microenvironment. (PD-1/PD-L1&CTLA-4) Representative Dual ICIs include Cadonilimab.

The biological significance of PD-1 was elucidated following the discovery of its ligands PD-L1/PD-L2 (12, 13). CTLA-4, an immune checkpoint molecule highly expressed on activated T cells and Tregs, was first functionally characterized by Dr. James Allison’s team. It suppresses T cell proliferation and IL-2 production through competitive binding with B7 ligands (14). Although both anti-CTLA-4 and anti-PD-1/PD-L1 therapies restore T cell function by blocking inhibitory signals, their mechanisms differ fundamentally: CTLA-4 primarily modulates CD4^+^ T cell activation and migration by regulating antigen-presenting cell (APC)-mediated CD28-B7 interactions (15–17), whereas PD-1 acts downstream of T cell receptor (TCR) signaling to reverse CD8^+^ T cell exhaustion without affecting clonal expansion (15, 18). This mechanistic complementarity provides a strong rationale for combination immunotherapy strategies. The history of therapies targeting PD-1/PD-L1 and CTLA-4 is summarized in **Figure 1**.

## 2 The evolving role of immunotherapy in OC

The efficacy of ICI monotherapy in recurrent OC remains suboptimal. ICI responses are typically associated with high tumor mutational burden (TMB-H, generally >10–20 mut/Mb), mismatch repair deficiency (dMMR), or microsatellite instability-high (MSI-H) status—features that enhance tumor antigen presentation and lymphocyte infiltration (19). However, OC is characterized by a low mutational burden (1–3.5 mut/Mb), with only 5–15% of cases exhibiting dMMR/MSI-H. Although dostarlimab and pembrolizumab have been approved in the US for dMMR/MSI-H/TMB-H tumors, this subgroup represents a small fraction of OC patients, significantly limiting the applicability of immunotherapy (20, 21).

The KEYNOTE-100 trial demonstrated that while the anti-PD-1 antibody pembrolizumab yielded an objective response rate (ORR) of <10% in advanced recurrent OC, a subset of responders exhibited durable clinical benefit lasting beyond 6 months (22). Similarly, the JAVELIN Ovarian 200 trial evaluated the anti-PD-L1 antibody avelumab in combination with pegylated liposomal doxorubicin, yet the combination achieved only a 13% response rate without significant survival improvement, with long-term benefit observed in only a minority of patients (23). Notably, certain OC histologic subtypes (e.g., clear cell carcinoma) display heightened sensitivity to immunotherapy (24), whereas the predominant high-grade serous and mucinous subtypes remain largely refractory (25).

The immunosuppressive TME of OC further complicates therapeutic efficacy. OC TME is characterized by low-to-moderate lymphocytic infiltration and abundant immunosuppressive myeloid cells (e.g., MDSCs, tumor-associated macrophages (TAMs)), which impair residual T-cell antitumor activity (26–28). Additionally, the “cold” tumor phenotype of OC—marked by low antigenicity and immune evasion mechanisms—limits ICI effectiveness (26). To overcome resistance, combinatorial strategies involving ICIs with chemotherapy, antiangiogenic agents, or PARP inhibitors have been explored, but no significant synergistic effects have been observed to date (25). Recently, dual immune checkpoint blockade (e.g., CTLA-4 plus PD-1/PD-L1 inhibitors) has gained attention due to its mechanistic complementarity, yet whether this approach can effectively overcome OC’s immunosuppressive barriers requires further validation. Future research should focus on novel combination strategies targeting TME reprogramming, enhanced antigen presentation, or myeloid cell modulation to expand the population benefiting from immunotherapy. To better illustrate the current progress in dual-ICI therapy, we summarize key clinical trial data in **Table 1**.

**Table 1:** Summary of key clinical trial data on bispecific antibody immunotherapy for advanced OC.

Clinical trial	Phase	N	Cohort description	Investigational drug/regimen	mPFS; months	mOS; months	ORR, %	Ovarian cancer classification	Author (year)
NCT02498600	II	100	Measurable disease, 1–3 prior regimens, and PFI < 12 months.	Intravenous nivolumab (every 2 weeks) or induction with nivolumab plus ipilimumab for 4 doses (every 3 weeks)	2.9 vs 3.9	21.8 vs 28.1	Any PD-L1 present: 2.0 vs 1.8	Persistent or recurrent EOC.	Zamarin D (2020) (29, 30),
NCT02834013	II	17	A prospective, multicenter (1,016 US sites), multi-cohort, single-arm phase II trial, had a histologically confirmed diagnosis of any subtype of NEOC	Patients were treated with ipilimumab at 1 mg/kg IV every 6 weeks and nivolumab at 240 mg intravenously every 2 weeks, with treatment administered continuously	Granulosa: 58.3; Carcinosarcomas: 50.7; Sertoli-Leydig: 30.4; yolk sac: 8.7;	42.5	25	NEOCs	Chae YK (2024) (31),
NCT03355976	II	44	Relapsed extra-renal CCC after at least one prior therapy (no prior immunotherapy)	Nivolumab (240mg IV every two weeks) vs nivolumab combination with ipilimumab (1mg/kg every six weeks)	2.2 vs 5.6	17 vs 24.6	14.3 vs 33	Ovarian CCC	Dizon DS (2024) (32),
NCT03699449	II	70	A multicenter, open-label, five-arm, uncontrolled, umbrella trial	HRD-positive: Arm 1: Olaparib 200mg bid + cediranib 30mg qd Arm 2: Olaparib 300mg bid + durvalumab 1500mg q4w HRD-negative: Arm 3 (PD-L1+): Durvalumab 1500mg q4w + chemotherapy (PLD/topotecan/weekly paclitaxel) for 6 cycles Arm 4 (PD-L1-): Durvalumab 1500mg q4w + tremelimumab 75mg q4w (4 doses) + chemotherapy (4 cycles) Arm 5: Durvalumab 1500mg q4w + tremelimumab 300mg (single dose) + weekly paclitaxel (60mg/m² D1,8,15 q4w for 4 cycles)*	5.62, 5.36, 3.68, 3.98, and 5.13	NR	50, 42.9, 20, 33.3, and 29.4	Platinum-resistant ovarian cancer(high-grade serous or endometrioid ovarian, primary peritoneal, or fallopian tube cancers)	Lee JY (2022) (33),
NCT03899610	II	45	Multicenter, two-arm, phase II trial, patients with stage IIIC-IVB ovarian cancer	In arm 1, patients received carboplatin at an AUC of five or six and an intravenous paclitaxel at a dosage of 175 mg/m2 on day 1, every 21 days for three cycles, along with durvalumab (1,500 mg for three cycles) and tremelimumab (75 mg for three cycles). In arm 2, patients received the same standard-of-care carboplatin and paclitaxel, with the addition of durvalumab (1,500 mg for three cycles) and a single dose of tremelimumab (300 mg in cycle 1). Arm 2 was initiated after the completion of arm 1.	15.8	NR	86.7	EOC with stages IIIC–IV disease	Park J (2023) (34, 35),
NCT03249142	Ib	70	Ib randomized trial	NACT + durvalumab (1125mg) alone (arm A) or with tremelimumab (75mg once at C2) (arm B)	25 vs 14	NR	NR	NR	Leary A (2024) (36),
NCT03026062	II	61	Randomized phase II trial	Patients were adaptively randomized to sequential arm: tremelimumab (3mg/kg q4 weeks x 4 doses) followed by durvalumab (1.5g IV q4 weeks for up to 9 doses) upon progression, or the combination arm: tremelimumab (1mg/kg IV plus durvalumab 1.5g IV q4wk for up to 4 doses followed by durvalumab monotherapy for up to 9 doses)	1.84 vs 1.87	10.61 vs 7.26	NR	High grade serous ovarian cancer	Hinchcliff E (2021) (37),
NCT05430906	II	17	An open-label, prospective, single arm, phase II study of evaluating the efficacy and safety of cadonilimab combined with chemotherapy as neoadjuvant treatment for advanced ovarian cancer	Patients received neoadjuvant therapy of cadonilimab plus platinum-taxane chemotherapy (3 to 4 cycles) followed by surgery. The primary endpoint was the R0 rate	NR	NR	94.1	High-grade serous carcinoma (94.1%)	Tang J (2024) (38),

PFS, Progression-free survival; OS, Overall survival; ORR, Objective response rate; PFI, Platinum-free interval; NACT, Neoadjuvant chemotherapy; NR, Not reported; CCC, Extra-renal clear cell cancer; EOC, Epithelial ovarian cancer; HRD, Homologous Recombination Deficiency; AUC, Area Under the Curve;

*Arm 5 was initiated after Arm 4 completion.

## 3 Advances in dual immune checkpoint inhibitor therapy for recurrent or platinum-resistant OC

Recent years have witnessed significant advancements in dual ICIs therapy for recurrent or platinum-resistant ovarian cancer (PROC), with emerging clinical evidence highlighting both its therapeutic potential and associated challenges. The Korean NCT03699449 trial (33) systematically evaluated efficacy differences among various immunotherapeutic regimens in 70 platinum-resistant patients. Notably, the combination of durvalumab (anti-PD-L1, 1500mg) with tremelimumab (anti-CTLA-4, 75mg) plus chemotherapy (n=18) demonstrated a superior ORR of 33.3% (95% CI: 13.3%-59.0%) compared to single-agent immunotherapy combined with chemotherapy (n=5). However, this regimen raised safety concerns, with grade 3/4 treatment-related adverse events (TRAEs) occurring in 66.7% of patients - substantially higher than other treatment groups (20%-47.1%). The investigators proposed that weekly low-dose paclitaxel administration might improve the safety profile. The NRG GY003 study (29, 30), involving 100 patients with recurrent OC (1–3 prior lines of chemotherapy and platinum-free interval <12 months), demonstrated that nivolumab (anti-PD-1) plus ipilimumab (anti-CTLA-4) significantly improved ORR (31.4% vs 12.2%, *P* = 0.034) and median progression-free survival (3.9 vs 2.0 months; HR = 0.53, *P* = 0.004) compared to nivolumab monotherapy. However, no statistically significant difference was observed in median overall survival (28.1 vs 21.8 months, *P* = 0.43). Importantly, biomarker analysis revealed no significant correlation between PD-L1 expression and treatment efficacy. The combination therapy maintained a manageable safety profile, with grade ≥3 TRAEs occurring in 49% of patients, consistent with previous reports.

The recently published phase Ib expansion study (39) demonstrated promising clinical outcomes with the novel dual immunotherapy combination of botensilimab (a next-generation multifunctional CTLA-4 inhibitor) plus balstilimab (PD-1 inhibitor) in 17 evaluable patients with recurrent platinum-resistant/refractory OC. Key efficacy data showed a median treatment duration of 3.2 months (range 0.9-19.6) and median follow-up of 8.8 months (range 2.0-29.2), with an ORR of 29% (5/17; 95% CI:13-53), including 4 confirmed partial responses and 1 unconfirmed complete response. The safety profile was favorable, with no reported cases of hypophysitis, myocarditis, or pneumonitis of any grade. TRAEs occurred at grade 1/2 in 94%, grade 3 in 24%, and grade 4 in 6% of patients, with no grade 5 events observed. Diarrhea/colitis was the most common grade 3/4 TRAE (18%). These findings suggest this dual checkpoint blockade combination achieves meaningful clinical activity with manageable toxicity in this heavily pretreated population, warranting further investigation in larger clinical trials to confirm its long-term efficacy and safety. A clinical study conducted by Hinchcliff et al. (37) investigated different administration strategies of CTLA-4 and PD-L1 inhibitors to evaluate their impact on progression-free survival (PFS) in platinum-resistant/refractory high-grade OC. The trial enrolled 61 platinum-resistant patients randomized to either sequential therapy (n=38; tremelimumab followed by durvalumab) or combination therapy (n=23; concurrent administration). Results demonstrated no statistically significant difference in median PFS between groups (*P* = 0.402). The sequential arm showed no objective responses, with 31.6% (12/38) achieving stable disease (SD), while the combination arm yielded partial responses in 8.7% (2/23) plus one SD case. Current data reflect outcomes in the high-grade serous ovarian carcinoma (HGSOC) subgroup, with enrollment ongoing for clear cell carcinoma patients.

In novel immunotherapy development, the bispecific antibody ubamatamab (targeting CD3 and MUC16) has shown therapeutic potential (40, 41). The ongoing first-in-human R16-ONC-3 study (REGN4018) evaluates ubamatamab monotherapy or combined with cemiplimab in recurrent advanced epithelial ovarian, primary peritoneal, or fallopian tube cancer patients (≥18 years, ≥1 prior platinum-based regimen, CA-125≥2×ULN) using a dose-escalation design. As an innovative bispecific antibody (anti-MUC16×CD3), ubamatamab represents a promising immunotherapeutic strategy, though efficacy data await further analysis as the trial continues recruitment. These studies not only validate the clinical potential of dual immune checkpoint inhibition in recurrent/resistant OCbut also provide critical insights for treatment optimization, safety management, and biomarker exploration. Larger phase III trials are warranted to confirm these findings and identify optimal beneficiary populations.

## 4 Current advances in neoadjuvant dual immune checkpoint inhibition for OC

### 4.1 Combination therapy strategies with single-target ICIs

Recent years have witnessed significant breakthroughs in clinical research investigating neoadjuvant immunotherapy combinations for advanced OC. The Ib phase randomized clinical trial presented at the 2024 ASCO Annual Meeting substantiated this progress (36). This study enrolled patients with unresectable stage IIIC/IV OC, demonstrating that neoadjuvant chemotherapy (NACT) combined with immunotherapy significantly improved complete cytoreduction rate (CC0), pathological complete response (pCR) rate, and PFS. Notably, while the dual immune checkpoint blockade (Group B, durvalumab + tremelimumab) showed no superior tumor immune microenvironment infiltration or survival outcomes compared to durvalumab monotherapy (Group A), the study identified pCR, CC0, and BRCA mutation status as independent predictors of long-term efficacy, rather than conventional immune-related biomarkers. These findings provide novel insights for precision medicine in OC, though the underlying mechanisms and clinical implications require further investigation.

The results suggest that NACT combined with dual ICIs (durvalumab + tremelimumab) may enhance antitumor effects through immune synergy, offering a potential therapeutic strategy for advanced epithelial ovarian cancer (EOC). The innovative KGOG 3046/TRUD study (NCT03899610) (34, 35) explored this dual immunotherapy-chemotherapy approach in the neoadjuvant setting. Initial 2023 results showed patients receiving durvalumab (1500mg) + tremelimumab (75mg) + NACT achieved 73.9% R0 resection and 17.4% pCR rates, with 12- and 24-month PFS rates of 63.6% and 45.0%, respectively. The 2025 final analysis further stratified 45 patients into multiple low-dose (n=23) and single high-dose (n=22) cohorts, revealing superior outcomes in the former for ORR (95.7% vs 77.3%, *P* = 0.048), chemotherapy response score (CRS) grade 3 response (39.1% vs 22.7%), and pCR rates (17.4% vs 4.6%). The entire cohort maintained 65.9% 12-month and 36.4% 30-month PFS rates. Safety analysis showed universal TRAEs occurrence, with rash being most common and neutropenia predominating grade 3–4 events. Beyond clinical validation, comprehensive biomarker analysis identified predictive potential for PD-L1 expression, mutational signature 3, and extracellular matrix components, providing critical evidence for precision immunotherapy approaches.

### 4.2 Dual-specificity immune checkpoint blockade as monotherapy

Cadonilimab (AK104), a novel tetravalent bispecific antibody targeting both PD-1 and CTLA-4, has demonstrated enhanced tumor-targeting capability through its unique structural design and is currently approved in China for metastatic/recurrent cervical cancer (42). The ongoing phase II NCT05430906 trial (38) is evaluating its efficacy and safety in combination with NACT for advanced OC, with R0 resection rate as the primary endpoint. Among 17 enrolled patients (94.1% high-grade serous carcinoma, 82.4% stage IVb), the regimen achieved an R0 resection rate of 66.7%, the ORR of 94.1% (including 1 complete and 15 partial responses), a pCR rate of 11.8%, and a CRS of 3 in 17.6% of patients. TRAEs occurred in 76.5% of patients, primarily thyroid dysfunction (23.5%) and myelosuppression (17.6%), with grade ≥3 events observed in 17.6% of cases including one grade 3 immune-related colitis (5.9%), all of which were manageable. These promising preliminary results support the potential clinical value of cadonilimab plus NACT in advanced OC though longer-term outcomes require further validation. In a separate case report by Zhang et al. (43), a PD-L1-positive (CPS = 10) OC patient who showed limited response to NACT plus bevacizumab subsequently achieved complete response with normalized CA-125 levels following cadonilimab maintenance therapy, suggesting that dual immune checkpoint inhibition may offer significant clinical benefit for HR-proficient/PD-L1-positive OC patients and providing important insights for optimizing treatment strategies in this population.

## 5 Clinical evaluation of dual immune checkpoint inhibition in OC subtypes

A phase II clinical trial (NCT03158064) (44) evaluated the efficacy of durvalumab plus tremelimumab in 29 patients with recurrent/refractory germ cell tumors, with only one ovarian primary case included according to the conference abstract. Although the overall treatment efficacy was limited (16-week PFS rate: 13.8%; median PFS: 1.4 months), one patient achieved sustained partial response (59% tumor reduction with PFS reaching 33 months), and two additional patients demonstrated tumor shrinkage exceeding 20%. Safety data showed grade 3–4 adverse events occurring in 21% of patients, suggesting potential benefit for specific patient subsets that warrants further exploration of biomarker-guided treatment strategies. These findings indicate that dual immune checkpoint inhibition may provide clinical benefit for certain germ cell tumor patients. Despite the modest overall efficacy, this study provides preliminary evidence supporting further investigation of immunotherapy in germ cell malignancies, including those of ovarian origin.

For clear cell carcinoma, updated results from the phase II trial (NCT03355976) (32) presented at ASCO 2024 demonstrated superior efficacy of nivolumab plus ipilimumab in recurrent extrarenal clear cell carcinomas (n=44). The combination arm showed significantly higher ORR (33.3% vs 14.3%) and median PFS (5.6 vs 2.2 months) compared to monotherapy, with a manageable safety profile (47% grade 3–4 AEs; no new safety signals). These data support the clinical utility of dual ICIs in ovarian clear cell carcinoma and other extrarenal variants. Collectively, these studies provide foundational evidence for immunotherapy in rare OC subtypes while underscoring the imperative for precision patient selection. Larger prospective trials are needed to validate these observations and optimize therapeutic strategies.

Clinical research on dual immune checkpoint blockade as first-line therapy for advanced OC remains limited. A pivotal phase III trial (NCT07002346) (45) registered on ClinicalTrials.gov is evaluating the efficacy of iparomlimab and tuvonralimab (QL1706) plus bevacizumab versus standard chemotherapy (paclitaxel plus carboplatin) in advanced ovarian clear cell carcinoma. Iparomlimab and tuvonralimab is an innovative bifunctional combination antibody comprising a recombinant humanized IgG4 monoclonal antibody targeting PD-1 and a recombinant humanized IgG1 monoclonal antibody targeting CTLA-4. This randomized, open-label, parallel-assignment phase III study plans to enroll 226 patients who will be randomized to receive either QL1706 plus bevacizumab or standard paclitaxel/carboplatin chemotherapy. The primary endpoint is PFS, with investigator-assessed overall survival (OS) as a key secondary endpoint. This trial will provide crucial evidence for immunotherapy combinations in advanced ovarian clear cell carcinoma, though data remain pending. Overall, investigation of dual immune checkpoint inhibition in first-line OC treatment is still in its early stages. Future research should focus on optimizing combination strategies, assessing safety profiles, and identifying predictive biomarkers to better select potential beneficiaries.

## 6 Conclusions and future perspectives

### 6.1 Future perspectives

Despite the demonstrated potential of ICIs in oncology, their clinical efficacy in OC remains suboptimal, characterized by low to modest objective response rates in most unselected patient cohorts (46, 47). This limitation stems from a confluence of factors intrinsic to OC biology, including a profoundly immunosuppressive TME, a relatively low TMB, the absence of validated predictive biomarkers, and both primary and acquired resistance mechanisms (38, 39). Given the remarkable inter- and intra-tumoral heterogeneity of OC, a paradigm shift is urgently required. Future therapeutic advancements hinge on a dual-pronged strategy: the development of mechanistically rational combination therapies and the implementation of sophisticated, biomarker-driven patient selection.

The future of ICIs in OC lies in strategic combinations designed to reverse immunosuppression and trigger robust anti-tumor immunity. The synergy between ICIs and PARPi extends beyond simple cytotoxicity. Evidence suggests that PARPi can upregulate PD-L1 expression on tumor cells, creating a biological rationale for combined blockade (48). In homologous recombination (HR)-deficient models, PARPi-induced DNA damage leads to the cytoplasmic accumulation of double-stranded DNA, which activates the cGAS-STING pathway (49, 50). This activation promotes the production of type I interferons and other inflammatory cytokines, thereby enhancing the recruitment and activation of CD8^+^ T-cells and converting immunologically “cold” tumors into “hot” ones (51–53). Clinical trials such as MEDIOLA (NCT02734004) have explored this combination, showing promising efficacy in BRCA-mutated OC (54). The combination of ICIs with antiangiogenic drugs like bevacizumab represents another rational approach. Beyond its role in inhibiting neovascularization, bevacizumab promotes vascular normalization, which alleviates tumor hypoxia and improves the perfusion and intratumoral infiltration of cytotoxic T lymphocytes (55). Concurrently, it counteracts VEGF-mediated immunosuppression by reducing the recruitment of MDSCs and Tregs within the TME (56). The phase III IMagyn050 trial, while not meeting its primary endpoint in the intent-to-treat population, provided insights into the potential of this combination and underscored the need for biomarker identification (57).

The profound heterogeneity of OC demands a paradigm shift toward precision medicine, underscored by the failure of single-agent ICIs and inconsistent combination trial outcomes that highlight the insufficiency of PD-L1 as a standalone biomarker. Therefore, developing and validating integrated, multi-parametric biomarker panels is a critical future direction. Key components should encompass: (i) immunohistochemical profiling, including quantitative and spatial analysis of tumor-infiltrating lymphocytes (TILs), particularly CD8^+^ T cells in the tumor core and invasive margin, which has been consistently associated with improved clinical outcomes (58); (ii) genomic and transcriptomic signatures, such as TMB, BRCA1/2 mutational status, and genomic scarring patterns (e.g., loss of heterozygosity), to identify tumors with elevated neoantigen burden and inherent genomic instability (59, 60), complemented by bulk and single-cell RNA sequencing to delineate the functional state of the TME and classify it into distinct phenotypes such as “immune-inflamed,” “immune-excluded,” or “immune-desert” (61, 62); and (iii) advanced integrative analytics that leverage machine learning algorithms to synthesize these disparate data streams—genomics, transcriptomics, proteomics, and digital pathology—to deconvolute OC complexity and generate predictive signatures for identifying patients most likely to achieve long-term benefit from immunotherapy (63).

Therefore, the next generation of clinical trials must be prospectively designed with the dual objective of evaluating the efficacy of rational drug combinations and rigorously validating sophisticated biomarker panels. This integrated strategy, which couples mechanistically grounded regimens with precision patient selection, is indispensable for unlocking the full therapeutic potential of ICIs in OC.

### 6.2 Conclusions

In summary, bispecific antibody-based immunotherapy represents a promising strategy for OC, particularly in platinum-resistant or recurrent cases. Early-phase clinical trials have demonstrated preliminary efficacy of agents simultaneously targeting dual immune checkpoints (e.g., PD-1/CTLA-4). However, challenges such as treatment-related toxicity, tumor heterogeneity, and the immunosuppressive microenvironment remain. Future success will depend on improved toxicity management, innovative combination therapies, validated biomarker development, and continued engineering of more effective and safer bispecific antibody constructs. Through collaborative and multidisciplinary efforts, the full therapeutic potential of ICP inhibition in OC may ultimately be realized.
